# Supplementary material for: Rootstock–Scion Exchanging mRNAs Participate in Watermelon Fruit Quality Improvement
Source: Int J Mol Sci. 2025 May 27;26(11):5121. doi: 10.3390/ijms26115121 (PMC12154523; doi:10.3390/ijms26115121)
Supplement: Supplementary file 1 [file ijms-26-05121-s001.zip › Supplementary figure S1.pdf]

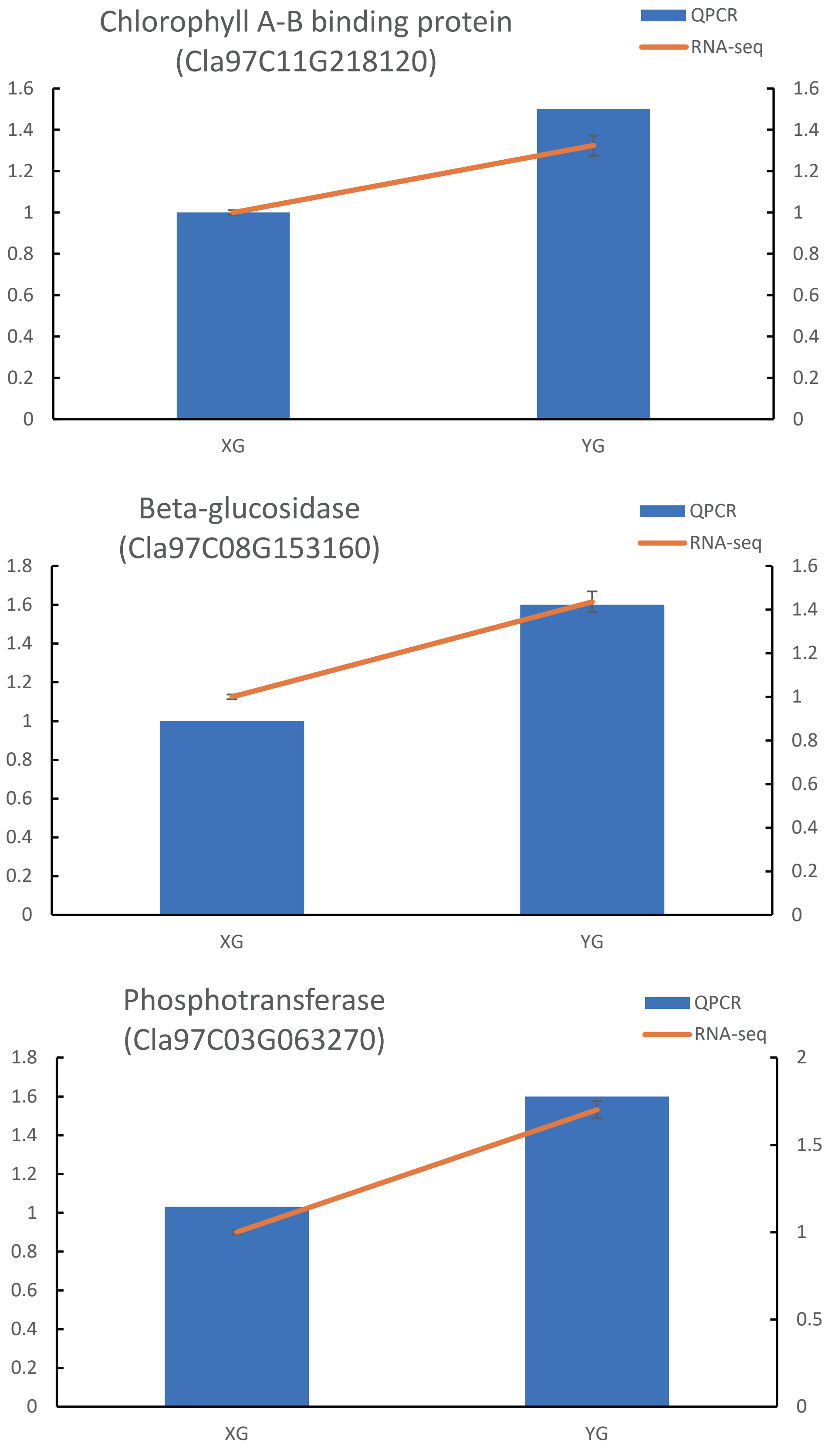

Supplementary Figure S1 RT-PCR of the mb-mRNAs to validate data reliability.
RT-PCR analysis and mb-mRNAs data of 4 genes (Cla97C11G218120, Cla97C08G153160, and Cla97C03G063270). The RT-PCR data are shown in the blue column, while the mb-mRNAs data are represented by the orange line.
